# Berry Quality and Anthocyanin Content of ‘Consort’ Black Currants Grown under Artificial Shade

**DOI:** 10.3390/plants10040766

**Published:** 2021-04-14

**Authors:** Eric Wolske, Laura Chatham, John Juvik, Bruce Branham

**Affiliations:** 1 Department of Crop Science, University of Illinois, Urbana, IL 61801, USA; ewolske2@illinois.edu (E.W.); juvik@illinois.edu (J.J.); 2 Department of Agronomy and Plant Genetics, University of Minnesota, St. Paul, MN 55108, USA; chatham@umn.org

**Keywords:** shade, black currant, *Ribes nigrum*, agroforestry, berry, anthocyanins

## Abstract

The effect of artificial shade on berry quality parameters for the field-grown black currant cultivar ‘Consort’ were investigated over two growing seasons in Urbana, Illinois. Four shade treatments reduced photosynthetically active radiation (PAR) from 37 to 83%. Shade had no effect on soluble solids in up to 65% PAR reduction but decreased 11% in 83% shade in one of two years. Shade increased titratable acidity up to 23% in both years. The effect of shade on anthocyanin content revealed greater variation between years than treatments. Shade influence on anthocyanin content was only observed in 2017, when cyanidin derivatives decreased 13–14% from open-sun to 83% shade. Shade did not affect delphinidin derivatives in either year. Environmental factors other than artificial shade may impact black currant berry quality in an understory environment. The results of our study indicate that black currants can maintain berry quality with PAR reductions up to 65%, but some berry quality parameters may decrease when PAR reductions exceed 65% of full sun.

## 1. Introduction

As global population continues to rise, agricultural production systems need to increase in efficiency to ensure food security and to provide ecosystem services. Agroforestry and multifunctional woody polycultures are agricultural systems designed to have vertical layers of production that combine a canopy of nut, fruit, and timber crops with an understory of fruit, nut, grain, or forage crops [1]. Similarly, agrivoltaics have been proposed as a form of agriculture where crops are grown under solar panels, creating environments where both food and energy can be harvested from the same area [2,3]. Urban agriculture is growing in the developed world as a way to take advantage of open city space to produce food locally while creating more positive green spaces in the built environment [4,5]. These innovative systems with multilayered production all contain an agriculturally important understory crop. For these agricultural systems to gain leverage, the quality of the crops produced in the understory environment needs to be better understood. 

Research into flavonoid biosynthesis in response to light and temperature has been explored in many fruit crops including grapes, coffee, strawberries, and blueberries with results affected by environment, temperature, and species. Further, some shade-adapted fruit species show minimal flavonoid response to environmental factors and are instead controlled by spatiotemporal regulation at different stages of fruit development [6]. Total anthocyanins in grapes were unaffected by shading but individual anthocyanins responded to shading [7]. In raspberries, blackberries, and strawberries, shade was found to increase polyphenols and acidity while sugar remained largely unaffected [8]. Coffee berries grown under shade trees or artificial shade had increased cupping quality and flavor, with larger, heavier berries produced [9,10]. The benefit of shade is strongly influenced by the environment and climate. Coffee quality was reduced when grown under shade at higher elevations [11]. 

Black currant (*Ribes nigrum* L.) is a berry crop that has agricultural potential in understory environments. The nutritious berry has high levels of ascorbic acid (Vitamin C) and flavonoids, both contributing to elevated antioxidant levels even when compared to other antioxidant-rich berries [12,13,14,15]. The major flavonoids are the anthocyanins cyanidin-3-*O*-glucoside, cyanidin-3-*O*-rutinoside, delphinidin-3-*O*-glucoside, and delphinidin-3-*O*-rutinoside [16,17,18]. Currants occur naturally in understory environments and are known to produce and grow well under shaded conditions [19,20,21]. The growth and productivity of black currants was maintained in up to 65% shade as found by [22]. Previous research on black currant berry quality in response to shade has shown a reduction in berry sucrose and glucose sugars, with an increase in citric acid in deep shade [19]. Shading also affects black currant fruit firmness, flavor, and secondary metabolites [20].

An effective polyculture system will require understory crops that can generate high quality produce under partial shade. Black currants have potential as an understory crop, with healthy, marketable, good-yielding fruit and shade-tolerant plant growth. However, there is limited research on the effects of shade on black currant berry quality, particularly in North American environments. Before black currants can be considered as a potential understory crop, it is essential to understand if acceptable levels of berry quality can be maintained in these environments. The aim of this study was to investigate the role of shade on black currant berry physical and biochemical quality characteristics grown in the Midwest United States.

## 2. Results

### 2.1. Berry Physical Properties

**Figure 1:**
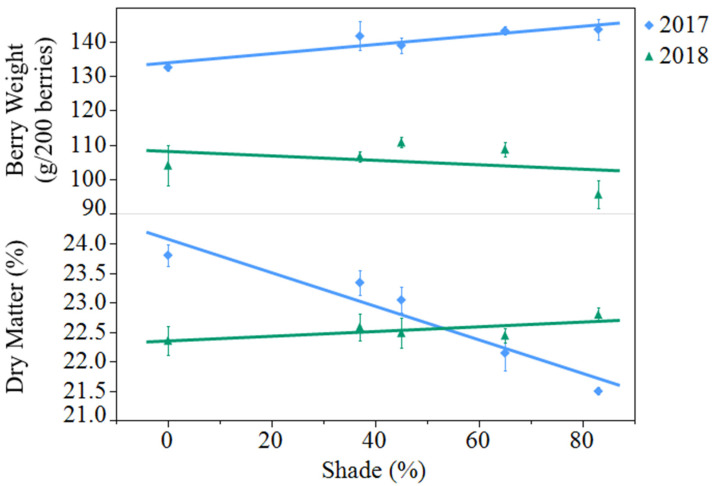
Effect of artificial shade treatment on berry fresh weight and dry matter of black currants (*Ribes nigrum* c.v ‘Consort’) grown in Urbana, IL in 2017 and 2018. Data are presented as the mean ± standard error of n = 4 replicates. Regression equations (where x = percent shade) were as follows: berry weight (2017) = 134.0 + 0.1331x, r^2^ = 0.38, *p* value = 0.0040; berry weight (2018) = 108.1 − 0.0648x, r^2^ = 0.05, *p* value = 0.3360; berry dry matter (2017) = 24.1 − 0.0286x, r^2^ = 0.77, *p* value < 0.0001; berry dry matter (2018) = 22.4 + 0.0040x, r^2^ = 0.09, *p* value = 0.2123.

Average berry fresh weight decreased from 2017 to 2018, from 140 to 105 grams per 200 berries (*p* < 0.0001). In 2017, shading resulted in an 8% increase in berry weight from open-sun to 83% shade, while shade did not affect berry weight in 2018 (Figure 1). Berry dry matter was not different between years but saw a significant year by treatment interaction (*p* < 0.0001). In 2017, there was a 10% decrease in dry matter from open-sun to 83% shade, while shade had no effect in 2018. 

### 2.2. Berry Chemistry

**Figure 2:**
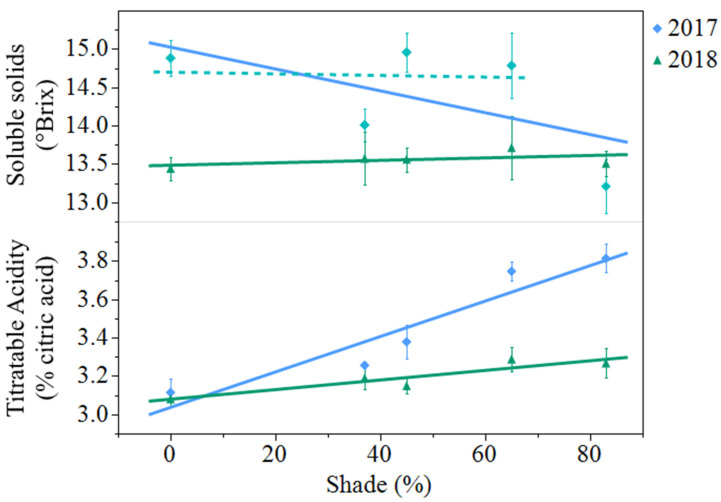
Artificial shade treatment effects on soluble solids and titratable acidity in juice of black currants (*Ribes nigrum* c.v ‘Consort’) harvested in Urbana, IL in 2017 and 2018. Data are presented as the mean ± standard error of n = 4 replicates. Regression equations (where x = percent shade) were as follows: soluble solids (2017) = 15.0 − 0.0143x, r^2^ = 0.22, *p* value = 0.0386; soluble solids (2017) regression without 83% shade treatment included in the model was not significant *p* value = 0.8852. Soluble solids (2018) = 13.5 + 0.0016x, r^2^ = 0.01, *p* value = 0.6961; titratable acidity (2017) = 3.04 + 0.0092x, r^2^ = 0.77, *p* value < 0.0001; titratable acidity (2018) = 3.08 + 0.0025x, r^2^ = 0.31, *p* value = 0.0101.

Soluble solids decreased from 2017 to 2018 (*p* = 0.0007). In 2017, soluble solids decreased 11% from open-sun to 83% shade. However, when the same regression model was analyzed with 83% shade excluded from the model, shade had no effect on berry soluble solids (Figure 2). In 2018, shade levels had no effect on soluble solids. Titratable acidity decreased from 3.46% citric acid in 2017 to 3.19% citric acid in 2018 (*p* < 0.0001). Titratable acidity increased from the open-sun to 83% shade by 23% in 2017 and by 6% in 2018. (Figure 2).

### 2.3. Anthocyanins

**Figure 3:**
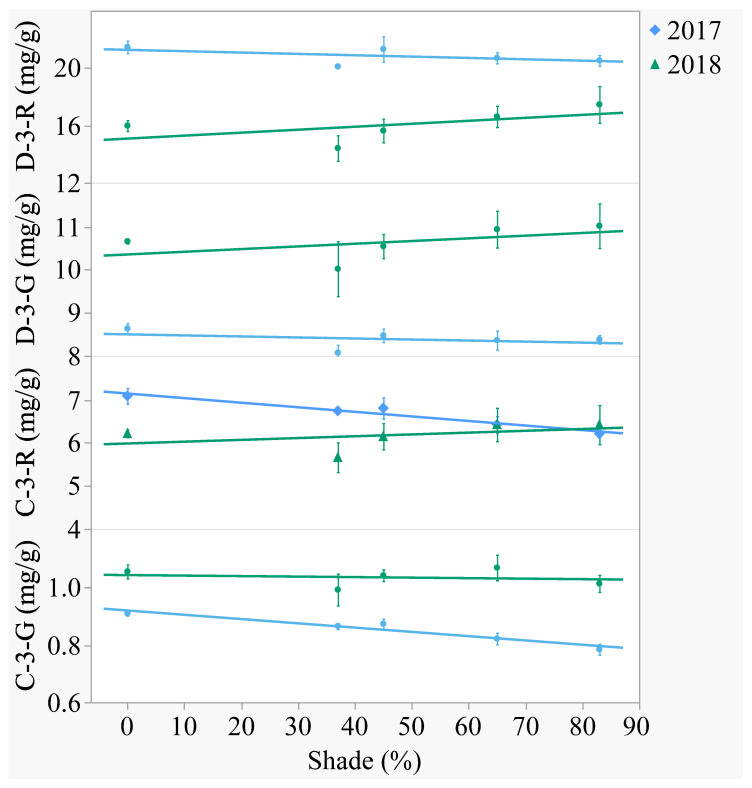
Artificial shade treatment effects on the four major anthocyanins of lyophilized black currant berries (*Ribes nigrum* c.v ‘Consort’) grown in Urbana, IL in 2017 and 2018. Anthocyanins measured were delphinidin-3-*O*-rutinoside (D-3-R), delphinidin-3-*O*-glucoside (D-3-G), cyanidin-3-*O*-rutinoside (C-3-R), and cyanidin-3-*O*-glucoside (C-3-G). Data are presented as the mean mg/g dry weight ± standard error of n-4 replicates. Regression equations (where x = percent shade) were as follows: D-3-R (2017) = 21.27 − 0.0095x, r^2^ = 0.07, *p* value = 0.2688; D-3-R (2018) = 15.07 − 0.0207x, r^2^ = 0.10, *p* value = 0.1720; D-3-G (2017) = 8.50 − 0.0024x, r^2^ = 0.04, *p* value = 0.4015; D-3-G (2018) = 10.35 − 0.0062x, r^2^ = 0.04, *p* value = 0.3743; C-3-R (2017) = 7.15 − 0.0106x, r^2^ = 0.49, *p* value = 0.0005; C-3-R (2018) = 5.99 + 0.0042x, r^2^ = 0.03, *p* value = 0.4451; C-3-G (2017) = 0.92 − 0.0015x, r^2^ = 0.66, *p* value < 0.0001; C-3-G (2018) = 1.04 − 0.0002x, r^2^ = 0.01, *p* value = 0.7626.

**Figure 4:**
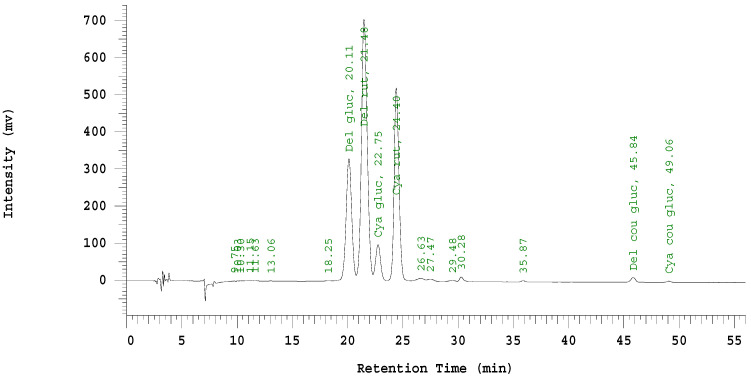
HPLC chromatogram (520 nm) of 1% HCl methanol-extracted anthocyanins from lyophilized ground black currant fruit.

Of the four major anthocyanins, delphinidin was the aglycone present in greatest content (Figure 3 and Figure 4). In both years, delphinidin-3-*O*-rutinoside had the highest content and cyanidin-3-*O*-glucoside had the lowest content when averaged across treatments. Anthocyanins differed between years, with the -3-*O*-rutinoside glycosides decreasing from 2017 to 2018 (*p* < 0.0001) and the -3-*O*-glucoside glycosides increasing (*p* < 0.0001). Out of the 2 aglycones, only cyanidin was affected by shade. In 2017, cyanidin-3-*O*-rutinoside decreased 13% and cyanidin-3-*O*-glucoside decreased 14% from the open-sun to 83% shade. In 2018, there was no effect of shade on anthocyanin content.

## 3. Discussion

Our study included a difference in shade netting color (black and white) and harvest dates between treatments (45%, 65%, and 83% harvested 4 days later than other treatments in 2017). The impact of shade netting density and color on *Vaccinium* crop environment and berry quality was explored by [23], with 25% white and 90% black shade netting included in their study. Black shade netting had a higher blue to red light ratio compared to white shade netting, which was similar to full sun. Red to far red ratios were similar for all shade netting studied. Similar to our results, in 2017, harvest dates in blueberries were delayed by 20 days under 90% black shade netting, while bilberries were unaffected. Different harvest dates in black currants were also explored by [24], who found an increase in sugars and anthocyanins and a decrease in total acids as fruit went from underripe red to overripe and dropping from the plant. However, in our study all plants were harvested at peak ripeness before fruit drop. 

Our study also utilized different fertilizer sources using an organic nitrogen source in 2017, switching to urea in 2018. When comparing organically and conventionally grown black currant berries, Ref. [25] found no differences in biochemical quality of the fruits. Based upon the results of [25], our use of different fertilizer inputs between the two seasons should have little impact on our results.

### 3.1. Berry Physical Properties

Berry weight can be an important factor in determining end-use products and harvest methods. Larger berries are easier for hand-harvest and can provide a better product for fresh markets, while smaller berries are better suited for machine-harvest and processing. Our results indicate that shading has minimal impact on berry weight and may increase berry weight in some years, an important factor in the fresh berry market and for cultivars with small berry size like ‘Consort’. The berry weights in our trial were similar or larger than reported by [15], who found the average berry weight for ‘Consort’ to be 112 grams per 200 berries when grown in Willamette Valley, Oregon. Our results in 2017 confirmed the results of [19], who found lower black currant berry weight in the control than in shaded treatments. However, our results in 2018 were consistent with results of [21], who found no difference between 50% shaded black currant fresh berry weight and open-sun. Berry dry matter decreased with shading in one of two years. Environmental factors such as soil moisture through the berry filling period may have a greater effect on berry dry matter than shading alone and should be further explored. 

### 3.2. Berry Chemistry

The sugar level and acidity of berries can be a major factor in black currant end-use and for a favorable juice product. Our results were promising, as soluble solids were unaffected by shade up to 65% in 2017 and up to 83% in 2018. The effect of shade on berry soluble solids was not as pronounced as observed by [19], who found that black currants grown in full sun had higher soluble solids than shaded currants. Our results were similar to [8], who found no relationship between shade and sugar content in raspberries, with only limited effects of shade on sugar content in blackberry. In *Vaccinium* species, blueberry total soluble solid content was reduced under 90% black shade compared to 15–25% colored shade, while bilberries had minimal differences [23]. Levels of berry soluble solids reported here were within the range of values found by [26] in North and South Sweden, but lower than the majority of cultivars tested by [27,28] in Southeastern Norway. 

Titratable acidity increased with shading in both years. Differences between years were confirmed by [29], who found significant seasonal variability from 1972–2007, with total solar radiation strongly correlated to seasonal ascorbic acid content compared to total precipitation or average daily temperature. Titratable acidity, even in 83% shade, was within the range of values reported by [27] and was much lower than the values reported by [26,28]. Previous research found an increase in citric acid production with shading in strawberry but no differences in blackberry and raspberry, while tartaric and malic acid increased with shading in raspberry and blackberry but was unaffected in strawberries [8]. Our observed values represent a good source of sugars and a balanced acidity, with the control and intermediate shade treatments yielding higher sugar and lower acidity; however, the reduced sugar observed in berries grown under 83% shade in 2018 may diminish marketability. 

### 3.3. Anthocyanins

Anthocyanins are responsible for the expression of color in berries and are the major source of antioxidant capacity [30], accounting for 70% of black currant antioxidant capacity [16]. Anthocyanins contribute to the health-promoting properties associated with black currants, including antimicrobial and tumor growth suppressing properties and benefits to the cardiovascular, nervous, ocular, skeletal, and renal systems [13]. If shaded agroecosystems continue to be explored for black currant production, it is important to understand the effects of shade on black currant anthocyanin content and to maintain the level of these marketable, health-promoting phytochemicals. 

The chromatograms (Figure 4) confirmed previous research that over 97% of the anthocyanins present in black currant berries were the four major anthocyanins found in this experiment [18]. Delphinidin was the major anthocyanin across all treatments and the -3-*O*-rutinosides were the major glycosides, confirming results of [16,31]. Our results indicate greater variation between years than between shade treatments, with minimal difference in anthocyanin content for plants grown under shade compared to full sun. Polyphenolics vary with growing seasons and the anthocyanins in our study also showed significant annual variation [26]. In a summary of previous anthocyanin research in grapes, Ref. [32] reported that the effects of temperature and light exposure on anthocyanin production can be difficult to separate, with the cultivar, site, and season all having significant effects on anthocyanin production in grapes. Other studies on anthocyanin accumulation in grapes found that shade decreased 3’-hydroxylated anthocyanin (e.g., cyanidin), but increased 3’,5’-hydroxylated anthocyanins (e.g., delphinidin) [33]. In contrast, Ref. [20] found cyanidin and delphinidin responded similarly to shade treatments within a genotype.

In blackberry and strawberry, total anthocyanins increased with shading, while in raspberry total anthocyanin production was maximized with 50% shade with lower anthocyanin production at 30% or 90% shade. Of the anthocyanins, cyanidin-3-*O*-glucoside increased with shading in strawberry and blackberry and peaked at 50% shade in raspberry while cyanidin-3-*O*-rutinoside increased with shading in blackberry [8]. In *Vaccinium* species [23] found 90% shade had the highest amounts of anthocyanin compared to full sun and 25% red netting in bilberries, while blueberries showed an inverse response with a decrease in anthocyanin content under 90% black shade netting in both years and under 15–25% colored shade netting, but only in the first year. In the second year, the lack of difference was attributed to the plants acclimating to the lower light environment. 

The response of black currant cultivars to shade was tested by [20], who found a variable response by currant cultivars under 30% shade. Our results found minor changes to anthocyanin content from shading, with differences only occurring in 2017, at maximum shading, and only with the cyanidin-derived anthocyanins. While our plants were acclimated a year before berry quality was measured, the additional year of acclimation may explain the lack of treatment differences between anthocyanins in the final year of this study in 2018.

## 4. Materials and Methods

This study was conducted during the 2017 and 2018 growing seasons on the Woody Perennial Polyculture project site at the University of Illinois Fruit Farm in Urbana, Illinois (40.079227, −88.216004). Soil types present were a Flanagan series (fine, smectitic, mesic Aquic Argiudolls) and a Thorp series (fine-silty, mixed, superactive, mesic Argiaquic Argialbolls) (Web Soil Survey, Natural Resources Conservation Service, United States Department of Agriculture). The existing site had east–west orientation with a 5-year-old *Ribes nigrum* ‘Consort’ set at 1.2 m spacing between plants and 4.8 m spacing between rows. Soil tests confirmed adequate levels of phosphorous (70 mg/kg) and potassium (250 mg/kg), thus only nitrogen was applied. Plants were fertilized in both years at a rate of 112 kg N/ha. In 2017, organic certification was considered and turkey manure was applied but this was abandoned by 2018. In 2018, nitrogen was applied as urea. Powdery mildew was observed and treated as needed from mid-May to mid-August with foliar applications of 2.6% *v*/*v* horticultural oil (Ultra-Pure, BASF Corporation, NC, USA) in 2017, and with 2.6% *v*/*v* horticultural oil and 1% *v*/*v* potassium bicarbonate (MilStop, Bioworks, NY, USA) in 2018. Weeds were removed in a 1.2 m band around plants using light-tillage only in 2017 and glyphosate in 2018. Pruning was done during dormancy to select approximately four new 1-year stems and 8 productive older stems for an average of 10–12 stems per plant post-pruning.

Four artificial shade treatments were used along with a non-shaded control. Shade netting at nominal levels of 20% white, 30% black, 50% black, and 70% black (Dewitt Company, Sikeston, MO, USA) were placed over six currant plants. The shade netting was installed in 2016, a year before data collection, to allow the plants to fully acclimate to the light environments and flower bud genesis to occur under treatments. Shade cloth photosynthetically active radiation (PAR) values were measured during the 2016 growing season with LI-190 quantum sensors (LI-COR Environmental Division, Lincoln, NE, USA) and averaged to determine actual PAR reduction values of 37%, 45%, 65%, and 83%, respectively and are reported as such. The 20% white shade netting treatment was used because we were unable to locate black shade netting less than 30% black shading used. A gothic frame structure 3 m in width and 1.8 m height in the center and slanting down to 0.9 m at the edges was built using 1.2 cm metal conduit. The shade netting was installed in late spring before full flower break on April 13th in 2017 and April 14th in 2018. Shade netting was removed after leaf abscission in late November in all years. Experimental design was a randomized complete block with four blocks. Each treatment consisted of six plants, with the outer two plants serving as buffers and data collected from the center four plants. 

### Berry Measurements and Analyses 

Treatments were harvested by hand when an average of 95% of the berries in a plot were ripe. Berry ripeness was visually estimated as percent dark purple skins and softened berries per plant and was averaged by plot. The four center plants in each plot were harvested individually. In 2017, open-sun and 37% shade treatments were harvested June 27th while 45%, 65%, and 83% shade treatments were harvested July 1st and 2nd with an extra 4 days required to reach full 95% ripeness. In 2018, all treatments reached peak ripeness similarly and were harvested on July 6th. For more information on methods and results relating to the yield and phenology of this study consult [22]. Subsamples of 300 berries per bush were removed for analysis. From this subsample, 100 berries were weighed and placed in a drying oven at 50 °C for at least 96 hours and reweighed to calculate percent dry matter. The 200 remaining berries were weighed to estimate berry fresh weight and were frozen at −20 °C for up to one year. 

Laboratory berry chemical quality measurements were conducted with 50 grams of frozen berries per replicate. Thawed berries were macerated in a blender and pressed through a 1.40 mm standard testing sieve (Sargent-Welch Scientific Company, Rochester, NY, USA). Juice soluble solids were measured with an Atago Digital Hand-held “Pocket” Refractometer PAL-1 (Atago USA, Inc., Bellevue, WA, USA). Titratable acidity was performed in duplicate by taking 6 g juice samples and adding 50 mL of water. Samples were titrated to a pH of 8.2 with 0.1 N NaOH using an Orion 350 PerpHect benchtop meter (Thermo Fisher Scientific, Waltham, MA, USA) with a wine must HI1048 pH electrode (Hanna Instruments, Inc., Woonsocket, RI, USA) and calculated as % citric acid according to [34]. 

Approximately 150 grams of frozen berries per plant were lightly macerated, frozen, and then lyophilized. The dried samples were ground using a coffee grinder and samples stored at −20 °C. From these samples, a 0.5 g subsample was vortexed with 5 mL of 1% HCl in methanol. The subsamples were then placed in a shaker set to 30 °C for 1 h. After shaking, the samples were centrifuged at 5000 rpm for 10 min, the supernatant filtered, and 0.5 mL placed in vials for analysis. A 20 μL aliquot of the sample was separated on an HPLC (Agilent 1100 series system; Santa Clara, CA, USA) with a diode array detector. A 100 mm × 4.6 mm × 2.7 µm Agilent Poroshell 120 SB-C18 Column was used. A mobile phase consisting of 2% (*v*/*v*) formic acid as solvent A and 100% acetonitrile as solvent B at a flow rate of 1 mL/min was used. A linear gradient beginning at 7% solvent B and increasing linearly to 13% over 15 min was used to separate individual anthocyanins. Column temperature was 30 °C and absorbance was measured at 520 nm. Peaks were integrated using ChemStation software (Agilent) and anthocyanin concentrations were determined using peak areas and standard curves of each major anthocyanin, cyanidin-3-*O*-rutinoside, cyanidin-3-*O*-glucoside, delphinidin-3-*O*-rutinoside, and delphinidin-3-*O*-glucoside (Polyphenols AS, Sandnes, Rogaland, Norway). Standard curves ranging from 1 to 1000 μg/mL were produced using Excel (Microsoft Corp., Redmond, WA, USA). To check for consistency between batches, 10% of the samples were run as duplicates. 

Analysis of variance was performed using JMP Pro (14.2.0, SAS Institute, Cary, NC, USA). The effects of year, shade, block, and year by shade interaction on the variables were analyzed using two-way analysis of variance (ANOVA). Year or year by treatment was significant (*p* < 0.05) for all variables so all data are reported by year. Variables were regressed against shade with least square regression and are presented as parameter means plus or minus standard error by treatment and year.

## 5. Conclusions

This is the first study in North America to explore the effects of light on black currant berry quality and one of only a few studies globally. Our results confirmed our hypothesis that black currants are a shade-tolerant crop that maintain high-quality fruit production with light reductions common to the understory environments of agroforestry and agrivoltaic growing systems. Titratable acidity was the only parameter that showed a consistent relationship with shade treatments, with an increase observed in both years. However, all treatments still resulted in lower titratable acidity values than values reported in native black currant production regions. Juice soluble solids, berry weight, berry dry matter, and individual anthocyanin content showed no consistent negative effects in up to 65% shade and negative effects of 85% shade were only observed as a reduction in juice soluble solids in one of two years. Anthocyanin content was largely unaffected by shade, with reduction from shade only observed in cyanidin in 2017. Finally, this study used shade netting to produce consistent levels of PAR reduction. Plants grown under natural tree shade are also affected by root competition for water and nutrients, an added stressor that may further impact berry quality. 

## Data Availability

The data presented in this study are available upon request from the corresponding author.
